# Polymorphonuclear myeloid-derived suppressor cells regulates immune recovery during HIV infection through PD-L1 and TGF-β pathways

**DOI:** 10.3389/fcimb.2024.1516421

**Published:** 2024-12-17

**Authors:** Zihua Wang, Yue Hu, Jing Song, Ping Ma, Huan Xia

**Affiliations:** ^1^ Department of Medical Oncology, Bethune International Peace Hospital, Shijiazhuang, China; ^2^ Department of Infectious Diseases, Tianjin Second People’s Hospital, Tianjin, China

**Keywords:** HIV, MDSC, PD-L1, TGF-β, immune recovery, immunological non-responders

## Abstract

**Background:**

Although MDSCs are widely recognized for their immunoinhibitory effects in pathological conditions, their function during HIV infection particularly within the mechanisms underlying incomplete immune recovery remains elusive.

**Methods:**

We conducted a cross-sectional study in which 30 healthy controls and 62 HIV-1-infected subjects [31 immunological non-responders (INRs) and 31 immunological responders (IRs)] were selected. The proportion of MDSCs was determined in each category of participants. Using flow cytometry and real-time PCR, immune regulatory molecules (including PD-L1, ARG1, iNOS, IL-10, TGF-β, and IDO) that are relevant for MDSCs activity were quantified. Furthermore, we investigated the impact of the blockade of PD-L1 and TGF-β pathways on MDSCs and their effects on CD4+ T-cells using *in vitro* functional experiments.

**Results:**

PMN-MDSCs are more abundant and are negatively correlated to CD4 counts in HIV-infected individuals. In addition, PMN-MDSCs suppress CD4+ T-cell proliferation and IFN-γ production in INRs. Furthermore, correlations were found between PD-L1 expression on PMN-MDSCs and PD-1+ CD4+ T-cells. TGF-β expression on PMN-MDSCs was likewise enhanced in INRs. Importantly, inhibiting both PD-L1 and TGF-β pathways had a synergistic impact on restoring CD4+ T-cell activity *in vitro*.

**Conclusions:**

PMN-MDSCs expansion inhibits CD4+ T-cell responses. We suggest that targeting PD-L1 and TGF-β pathways together may significantly improve immune recovery in INRs.

## 1 Background

In general, it is admitted that 10–40% of HIV-infected individuals on antiretroviral therapy (ART) may have inadequate CD4+ T-cell recovery despite virologic suppression (Yang et al., 2020). These people are referred to as immunological non-responders (INRs). On the contrary, another group of ART-treated individuals, known as immunological responders (IRs), have robust CD4+ T-cell recovery. In various studies conducted over the years, INRs have been defined by either a failure to achieve the specified CD4+ T-cell counts threshold (e.g., 350 or 500 cells/μL) or a certain percentage of CD4+ T-cell rise over baseline (e.g., 20% or 30%) (Yang et al., 2020). Several risk factors for inadequate CD4+ T-cells recovery have been reported, including lower nadir CD4+ T-cell counts, male sex, older age, longer duration of HIV infection, hepatitis B virus coinfection, and so on (Yang et al., 2020). To date, there is no treatment or adjunctive treatment for such a condition and the precise mechanisms responsible for incomplete immune recovery are elusive. Thus, it is urgent to identify possible therapeutic targets to enhance immunological recovery in INRs.

Myeloid-derived suppressor cells (MDSCs) are a heterogeneous population of innate immune cells, including myeloid progenitors and immature myeloid cells with potent immune suppressive activity (Veglia et al., 2018; Hegde et al., 2021). Under pathological settings, partial blocking of the differentiation of immature myeloid cells into mature cells can promote the expansion of the MDSCs (Dorhoi et al., 2019; Pawelec et al., 2019; Nourbakhsh et al., 2021; Vanhaver et al., 2021). In humans, MDSCs are characterized as CD11b+CD33+HLA-DR-/low and are often classified as either monocytic (M-MDSCs) or polymorphonuclear (PMN-MDSCs) subsets based on the presence of CD14 or CD15 (Bronte et al., 2016; Veglia et al., 2018). The hallmark of MDSCs is their capacity to inhibit T-cell and innate immune responses through various mechanisms, including the production of reactive oxygen species (ROS), inducible nitric oxide synthase (iNOS), indoleamine 2,3-dioxygenase (IDO), arginase1 (ARG1), interleukin 10 (IL-10), transforming growth factor beta (TGF-β), and the expansion of regulatory T-cells (Tregs) (Ostrand-Rosenberg et al., 2023).

Numerous studies indicate that MDSCs expansion occurs during HIV infection and is correlated with HIV disease progression (Ademe, 2020; Yaseen et al., 2021). MDSCs inhibit the proliferation of CD8+ and CD4+ T cells, which directly impairs their protective responses (Gama et al., 2012). MDSCs also stimulate the proliferation of IL-10, Tregs, and transiently induce programmed death-ligand 1(PD-L1) expression, resulting in T-cell exhaustion (Vollbrecht et al., 2012; Wang et al., 2016; Ademe, 2020). In such a context, the lack of protective T-cell response as well as the proliferation of the aforementioned Tregs and IL-10 facilitate persistent HIV infection (Ostrand-Rosenberg et al., 2023). Furthermore, pathologically expanded MDSCs can dampen anti-viral immune responses mediated by T-cells *via* indirect mechanisms, including inducing T-cell anergy by downregulating CD3ζ expression (Tumino et al., 2015) and induction of ARG1 (Qin et al., 2013). Interestingly, ART decreases the population of MDSCs in HIV-infected individuals within 6 weeks of therapy (Dross et al., 2017), but MDSCs increase again and stabilize at higher levels despite prolonged ART (Qin et al., 2013; Grutzner et al., 2018). Although significant advances and efforts put to decipher the specific roles of MDSCs in HIV/AIDS-related pathological conditions, their potential implications in the incomplete immune recovery process remain to be clarified.

In this study, we aimed to evaluate the role played by MDSCs in immune recovery and the potential mechanisms involved in such a process. To this purpose, we explored the frequency, phenotype, and function of circulating MDSCs in different groups of HIV-1 infected individuals, namely INRs and IRs, versus healthy controls.

## 2 Methods

### 2.1 Study subjects and samples

Subjects with chronic HIV-1 infection and undetectable viral loads, more than 2 years of ART, were enrolled at Tianjin Second People’s Hospital from December 2020 to May 2021. The participants were stratified either as INRs (CD4 <350 cells/μL) or as IRs (CD4 >500 cells/μL). Additionally, age-matched healthy controls (HCs) were recruited. Blood samples were collected; then, PBMCs were separated from the whole blood, and were subsequently stored at -80°C. The Tianjin Second People’s Hospital Ethics Committee authorized the study (2020-12). Each participant provided written informed consent prior to enrolment, which is in line with the Helsinki Declaration.

### 2.2 Flow cytometry

Cryopreserved PBMCs were thawed in RPMI 1640 media (Invitrogen, Carlsbad, CA, USA) with 10% fetal bovine serum (Gibco, Invitrogen, NY, USA). To determine the frequency and phenotypes of MDSCs, PBMCs were stained with the following antibodies: CD45-APC-H7, CD33-PE-Cy7, CD11b-BV605, HLA-DR-BV510, CD14-FITC, CD15-PerCP-Cy5.5 (BD Biosciences). To detect PD-L1 expression on MDSCs, PD-L1-PE (BD Biosciences) was added to the previously listed antibodies. T-cell phenotypes were stained with CD3-PerCP, CD4-PE-Cy7, CD8-APC-Cy7, and PD-1-BV605 (BD Biosciences) for 20 min at room temperature. Samples were then acquired and analyzed on FACS Canto Plus and LSRFortessa with Diva software (BD Biosciences). Fluorescence minus one controls or relative isotype controls were prepared to facilitate gating. Data were analyzed using the Flowjo 10 software (Tree Star Inc., Ashland, OR, USA).

MDSC subpopulation phenotypes were defined as follows: PMN-MDSC: CD45+HLA-DR–CD33+CD11b+CD14–CD15+, M-MDSC: CD45+HLA-DR–CD33+CD11b+CD14+CD15– (**Supplementary Figure 1**).

### 2.3 Cell sorting and T-cell suppression assays

PBMCs were isolated from fresh blood from 10 HIV-1-infected individuals by using Ficoll-Paque PLUS (GE Healthcare), and then PMN-MDSCs were sorted using CD15 MicroBeads and magnetic assisted cell sorting MS separation columns (Miltenyi Biotec, Bergisch Gladbach, Germany).

PBMCs, PMN-MDSCs depleted PBMCs (DEPL), and DEPL plus autologous sorted PMN-MDSCs were labeled with a cell tracker dye (1:4 ratio), carboxyfluorescein diacetate succinimidyl ester, (CFSE, Invitrogen, Carlsbad, CA, USA) at a final concentration of 5μM as recommended by the manufacturer. Then, cells in each group (PBMCs, DEPL, DEPL+MDSCs) were stimulated with anti-CD3/CD28 coated beads (1 μg/mL; BD Biosciences) in an incubator at 37°C and 5% CO_2_ for 4 days in R10. Finally, the cells were washed, stained with CD4-PE-Cy7 at day 5, and subjected to flow cytometry to assess T-cells proliferation.

For intracellular cytokine detection, these cells were incubated in R10 for 6 hours and stimulated with leukocyte activation cocktail (BD Biosciences), which contained phorbol 12-myristate-13-acetate (PMA), ionomycin, and brefeldin A. Cells were surface stained with CD3-BV650 (BD Biosciences), CD4-AF700 (Biolegend), and CD8-Percp-Cy5.5 (BD Biosciences), then further permeabilized with a IntraSure™ kit (BD Biosciences). Series of intracellular staining were performed with IFN-γ-BV605 (Biolegend), IL-2-APC (Biolegend), and TNF-α-PE-Cy7 (Biolegend). Samples were acquired and analyzed as described above.

For blocking experiments, T-cell suppression assays were performed using sorted MDSCs cocultured with autologous CD4+ T-cells in the presence or absence of inhibitors. Magnetic cell sorting CD4+ T-cell isolation kit (Miltenyi Biotec) was used to isolate autologous T-cells (CD4+ T-cells) from freshly isolated PBMCs. Flow cytometry revealed cell purity of >95% after all separations (data not shown). Prior to coculture, purified CD4+ T cells were labeled with CFSE, then mixed with sorted PMN-MDSCs (4:1 ratio) in the presence of anti-CD3/CD28 beads for 4 days. T-cells alone were used as controls. At the commencement of the experiments, either anti-PD-L1 antibody (10μg/mL), pure anti-human TGF-β neutralizing antibody (20 μg/mL), or anti-PD-L1 isotype control were administered to the co-cultured system. T-cell proliferation and intracellular cytokine detection were analyzed as described above.

### 2.4 Real-time PCR

TRIzol reagent was used to extract total RNA from PMN-MDSCs and DEPL. Then, RNA was reverse-transcribed to obtain cDNA using Superscript™ III First-Strand Synthesis system (Invitrogen, Carlsbad, CA, USA). cDNA product was set in a 20 μl amplification reaction, which contained 10 μl 2×SuperMix (Platinum SYBR Green qPCR kit; Invitrogen), 4 μl cDNA, 0.4 μl of each primer (10 μM), and 5.2 μl DEPC water. The reaction condition was as follows: 50 °C for 2 min and 95 °C for 5 min, then 50 cycles of 95 °C for 15s and 60 °C for 30s. ARG1, iNOS, IL-10, TGF-β, and IDO mRNA expressions were analyzed with real-time PCR using the following primers:

ARG1 Forward: 5′-CGCCAAGTCCAGAACCATAG-3′

Reverse: 5′-TCCCCATAATCCTTCACATCAC-3′;

iNOS Forward: 5′-AGATAAGTGACATAAGTGACCTG-3′

Reverse: 5′-CATTCTGCTGCTTGCTGAG-3′;

IL-10 Forward: 5′- GCCAAGCCTTGTCTGAGATG-3′

Reverse: 5′-AAGAAATCGATGACAGCGCC-3′;

TGF-β Forward: 5′-GACATCAACGGGTTCACTAC-3′

Reverse: 5′-GTGGAGCTGAAGCAATAGTT-3′;

IDO Forward: 5′-AGTTCTGGGATGCATCACCA-3′

Reverse: 5′-ACTGCAGTCTCCATCACGAA-3′.

The relative level of target mRNA expression was normalized to GAPDH (Forward: 5′-CCAGAACATCATCCCTGCCT-3′; Reverse: 5′-CCTGCTTCACCACCTTCTTG-3′) using the equation 2^−ΔΔCt^.

### 2.5 Plasma cytokine measurement

TGF-β levels in plasma samples were assessed using an ELISA kit (Invitrogen, CA, USA). Experiments were performed in accordance with the manufacturer’s instructions. The thresholds for detection were 0.098 ng/mL.

### 2.6 CD4+ T-cell counts and HIV-1 RNA measurements

CD4+ T-cell counts and plasma HIV-1 RNA were measured following an established procedure, which has been published previously (Xia et al., 2018).

### 2.7 Statistical analysis

Graphical presentation and statistical analyses were performed using GraphPad Prism version 8.0 (GraphPad Software, San Diego, CA, USA). Mann–Whitney *U* test or Student’s t-test (between two groups) and one-way ANOVA (for multiple groups) are used to compare continuous variables. Correlation between variables is estimated with Spearman’s nonparametric test. All tests are two-tailed, and *P* values < 0.05 is considered statistically significant.

## 3 Results

### 3.1 Characteristics of the study participants

Blood samples from 62 HIV-1-infected males, displaying undetectable HIV-1 viral loads as receiving ART for at least two years (31 INRs and 31 IRs), and 30 healthy controls were examined (**Table 1**). At the time of inclusion, the median CD4+ T-cell counts for INR and IR were 254 cells/μL and 736 cells/μL, respectively. INR had a lower median nadir CD4 counts than IR (35 vs. 309, *P* < 0.0001). Age, ART duration, and pre-ART viral loads did not significantly differ among the different HIV-1-infected groups.

**Table 1:** Characteristics of the study population.

	Healthy controls (*n* = 30)	HIV+, INR (*n* = 31)	HIV+, IR (*n* = 31)	*P* (INR vs. IR)
Male gender	30 (100%)	31 (100%)	31 (100%)	–
Age (years)	48 (41-55)	48 (37-60)	52 (35-58)	0.942
Current CD4+ T-cell count (cells/μL)	–	254 (197-310)	736 (616-812)	<0.0001
Nadir CD4+ T-cell count (cells/μL)	–	35 (16-72)	309 (197-246)	<0.0001
ART duration (years)	–	5.1 (4.5-6.5)	5.9 (4.8-7.6)	0.096
Current viral load (copies/mL)	–	undetectable	undetectable	–
Pretreatment viral load (copies/mL)	–	37,140 (17,294-87,200)	55,100 (19,700-12,000)	0.443

Data are presented as number (percentage) or median (interquartile ranges). HIV, human immunodeficiency virus; INR, immunological non-responders; IR, immunological responders; ART, antiretroviral therapy; –, not applicable. The non-parametric Mann–Whitney U test was used for statistical analysis.

### 3.2 PMN-MDSCs are expanded in HIV-1-infected individuals and correlated with CD4+ T-cell counts

**Figure 1:**
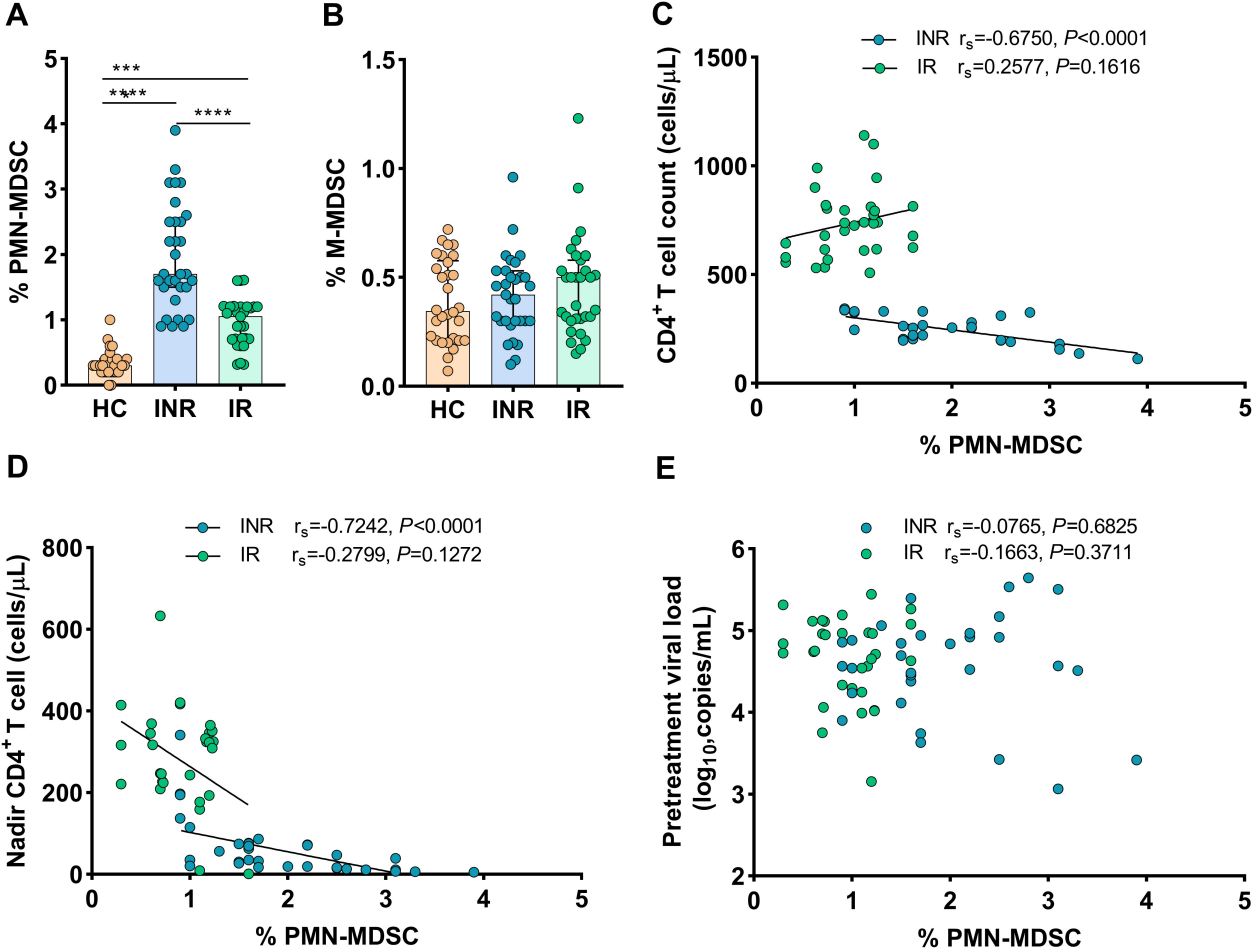
Frequency of MDSCs in HIV-infected individuals with different immune recovery status and its relation to CD4+ T-cell counts. The frequencies of PMN-MDSCs **(A)** and M-MDSCs **(B)** in three groups of study participants (HC *n* = 30, INR *n* = 31, and IR *n* = 31) were compared. Correlations between the frequency of PMN-MDSCs with CD4+ T-cell counts **(C)**, Nadir CD4+ T-cell counts **(D)**, and pretreatment viral loads **(E)**. The non-parametric Mann–Whitney *U* test was used for statistical analysis. Horizontal lines and error bars represent the median and interquartile ranges (IQR). Spearman’s nonparametric test was used for correlation analysis. HC, Healthy controls; INR, Immunological non-responders; IR, Immunological responders. ***P < 0.01, ****P < 0.0001.

To investigate the role of MDSCs in immunological recovery, we compared the proportions of MDSCs in HIV-1-infected individuals’ peripheral blood to that of HCs. As shown in **Figure 1A**, compared to INRs and IRs, lowest proportions of PMN-MDSCs were noted in HCs (all *P* < 0.0001). Interestingly, PMN-MDSCs were more abundant in INRs than in IRs (*P* < 0.0001, **Figure 1A**). On the other hand, we noted that the proportions of M-MDSCs were analogous across all groups (all *P* > 0.05, **Figure 1B**).

Then, we assessed the relationship between the proportions of MDSCs and HIV-1 disease progression (via IRs and INRs). In INRs, the proportions of PMN-MDSCs were shown to be inversely correlated to CD4+ T-cell counts (r_s_ = -0.6750, *P* < 0.0001, **Figure 1C**) and nadir CD4 counts (r_s_ = -0.7242, *P* < 0.0001, **Figure 1D**), but not to pretreatment HIV-1 viral loads (r_s_ = 0.0765, *P* = 0.6825) (**Figure 1E**). Conversely, in IRs, the proportions of PMN-MDSCs were not correlated to CD4+ T-cell counts (r_s_ = 0.2577, *P* = 0.1616, **Figure 1C**), nadir CD4 counts (r_s_ = –0.2799, *P* = 0.1272, **Figure 1D**), and pretreatment HIV-1 viral loads (r_s_ = 0.1663, *P* = 0.3711, **Figure 1E**). However, no correlations were found between M-MDSCs and disease progression markers such as nadir CD4 counts, CD4+ T-cell counts, or HIV-1 viral loads when data from both INRs and IRs were combined (data not shown). These findings indicated that PMN-MDSCs are potentially important in the immunological recovery process.

### 3.3 PMN-MDSCs inhibit CD4+ T-cell response in immunological non-responders

**Figure 2:**
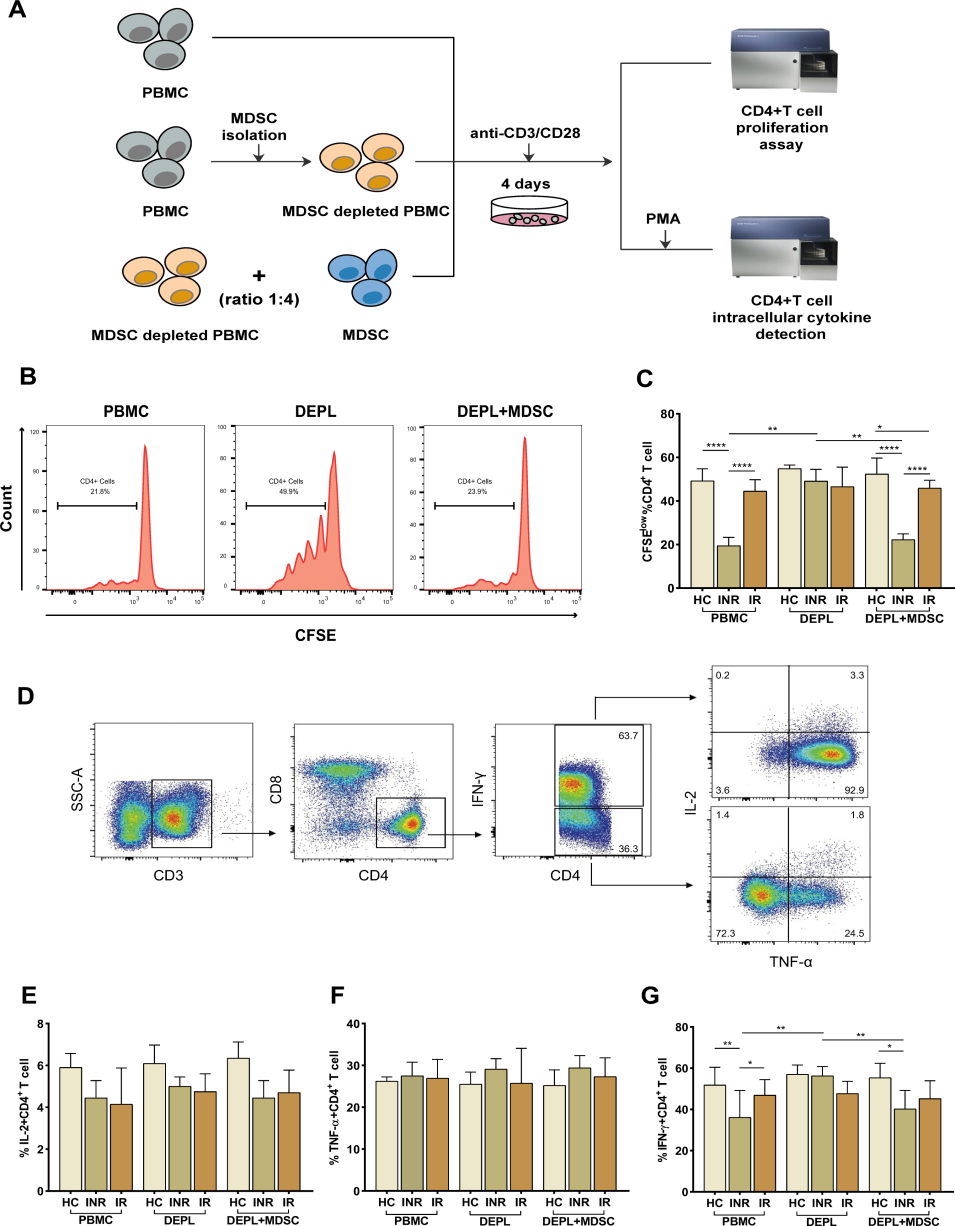
PMN-MDSCs inhibit autologous CD4+ T-cell proliferation and cytokines (IL-2, TNF-α, and IFN-γ) production in HIV INRs. Schematic representation of the experiments **(A)**. **(B)** Flow-cytometry histogram plots on CD4+ T-cell proliferation rates. **(C)** The percentage of CD4+ T-cell proliferating cells in PBMCs, PMN-MDSCs depleted PBMCs (DEPL), and DEPL plus PMN-MDSCs at a 1:4 ratio after 4 days of coculture with anti-CD3/CD28 beads. **(D)** Gating strategy for CD4+ T-cell, IL-2, TNF-α, and IFN-γ expression. IL-2 **(E)**, TNF-α **(F)**, and IFN-γ **(G)** levels expressed by CD4+ T cells in PBMCs, DEPL, and DEPL plus PMN-MDSCs at 1:4 ratio in the presence of PMA/ionomycin for 6 h. The results are shown as a median with an IQR. One-way ANOVA was used. *P < 0.05, **P < 0.01, ****P < 0.0001. ns, not significant. HC, Healthy controls; INR, Immunological non-responders; IR, Immunological responders.

To assess the suppressive activity of PMN-MDSCs, we examined their capacity to inhibit the proliferation of autologous CD4+ T-cells using CFSE dilution analysis. With the use of CD15 magnetic beads, pure PMN-MDSCs were isolated from freshly collected PBMCs. We examined T-cell proliferation after stimulating PBMCs, DEPL, and DEPL+MDSCs with anti-CD3/CD28 beads (ratio 1:4) for 4 days. The flow-chart for the experiment is shown in **Figure 2A**.

We found that PMN-MDSCs removal significantly enhanced CD4+ T-cells proliferation in INRs only. When PMN-MDSCs were re-added at a ratio of 1:4, CD4+ T-cells proliferation dropped down to a level comparable to that of the freshly collected PBMCs (**Figure 2C**). Compared to HC and INRs, MDSC-depleted INR PBMCs supplemented with autologous MDSCs have a statistically significant reduction in proliferation, (intermediate level, **Figure 2C**). Notably, in IRs, CD4+ T-cells proliferation remained relatively stable in PBMCs, MDSC-depleted PBMCs, and MDSC-depleted PBMCs supplemented with MDSCs.

Then, we assessed the inhibitory effects of MDSCs on cytokines release from autologous T-cells. Flow cytometry was used to detect intracellular IL-2, TNF-α, and IFN-γ after stimulation of CD4+ T-cells with PMA (**Figure 2D**). The frequency of IFN-γ–secreting CD4+ T-cells increased significantly after PMN-MDSCs depletion and declined after PMN-MDSCs addition to DEPL (**Figure 2G**). However, we did not find any increase in CD4+ T-cells generating IL-2 and TNF-α (**Figures 2E, F**). In HCs and IRs, no comparable outcomes were seen. In other words, the results revealed that PMN-MDSCs are not suppressive in HCs and doesn’t seem to have any effect on IRs.

### 3.4 PD-L1 expression on PMN-MDSCs correlates with PD-1 expressing CD4+ T-cell during HIV infection

Further studies were conducted on the mechanisms whereby MDSCs inhibit CD4+ T-cell response. Given that it is expressed on myeloid cells, PD-L1 is regarded as a crucial biomarker and a promising target in current immunotherapies (Yi et al., 2022). The interaction between PD-1 on T-cells and the inhibitory ligand PD-L1 expressed on myeloid cells was proven capable of inducing T-cell anergy (Bowers et al., 2014). In order to determine if the PD-L1/PD-1 axis is involved in the MDSCs-mediated inhibition of CD4+ T-cell function, flow cytometry was used to measure PD-L1 expression on PMN-MDSCs and PD-1 on CD4+ T-cells.

**Figure 3:**
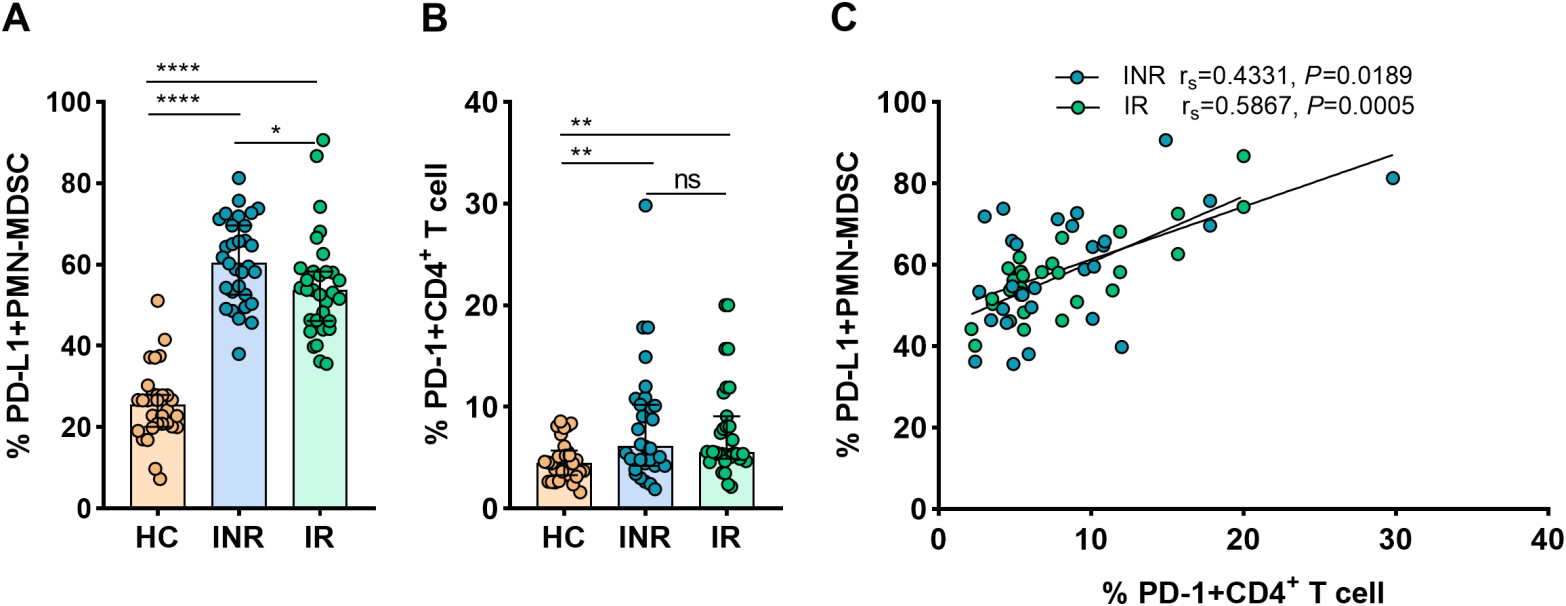
PD-L1 expression on PMN-MDSCs and its correlation with PD-1+CD4+ T-cells in HIV-infected individuals. PD-L1 expression on PMN-MDSCs **(A)** and PD-1 expression on CD4+ T-cells **(B)** in HCs and HIV-infected subjects with varying immunological recovery status. The results are shown as median (IQR). Correlation between PD-L1 expressing PMN-MDSCs and PD-1+CD4+ T cells in HIV-infected individuals **(C)**. HC, Healthy controls; INR, Immunological non-responders; IR, Immunological responders. The non-parametric Mann–Whitney *U* test was used for statistical analysis. Data are expressed as the median (IQR). Spearman’s nonparametric test was used for correlation analysis. *P < 0.05, **P < 0.01, ****P < 0.0001. ns, not significant.

**Figure 3A** demonstrates that the expression of PD-L1 on PMN-MDSCs was considerably higher in INRs and IRs than in HCs (*P* < 0.0001 for all comparisons). In INRs, the proportions of PD-L1+ PMN-MDSCs were significantly higher than in IRs (*P* < 0.05). In the same line, we observed that PD-1 expression on CD4+ T-cells was greater in INRs and IRs than in HCs (HCs vs. INRs, *P* < 0.01; HCs vs. IRs, *P* < 0.01; **Figure 3B**). However, between INRs and IRs, the proportions of CD4+ T-cells expressing PD-1 were statistically similar. Notably, we found a correlation between the expression of PD-L1 on PMN-MDSCs and PD-1 on CD4+ T-cells (INRs: r_s_ = 0.4331, *P* = 0.0189 and IRs: r_s_ = 0.5867, *P* = 0.0005; **Figure 3C**). Therefore, we speculate that PD-L1/PD-1 pathway may be crucial for the restoration of CD4 counts.

### 3.5 Increased TGF-β Expression on PMN-MDSCs in HIV-1 immunological non-responders

**Figure 4:**
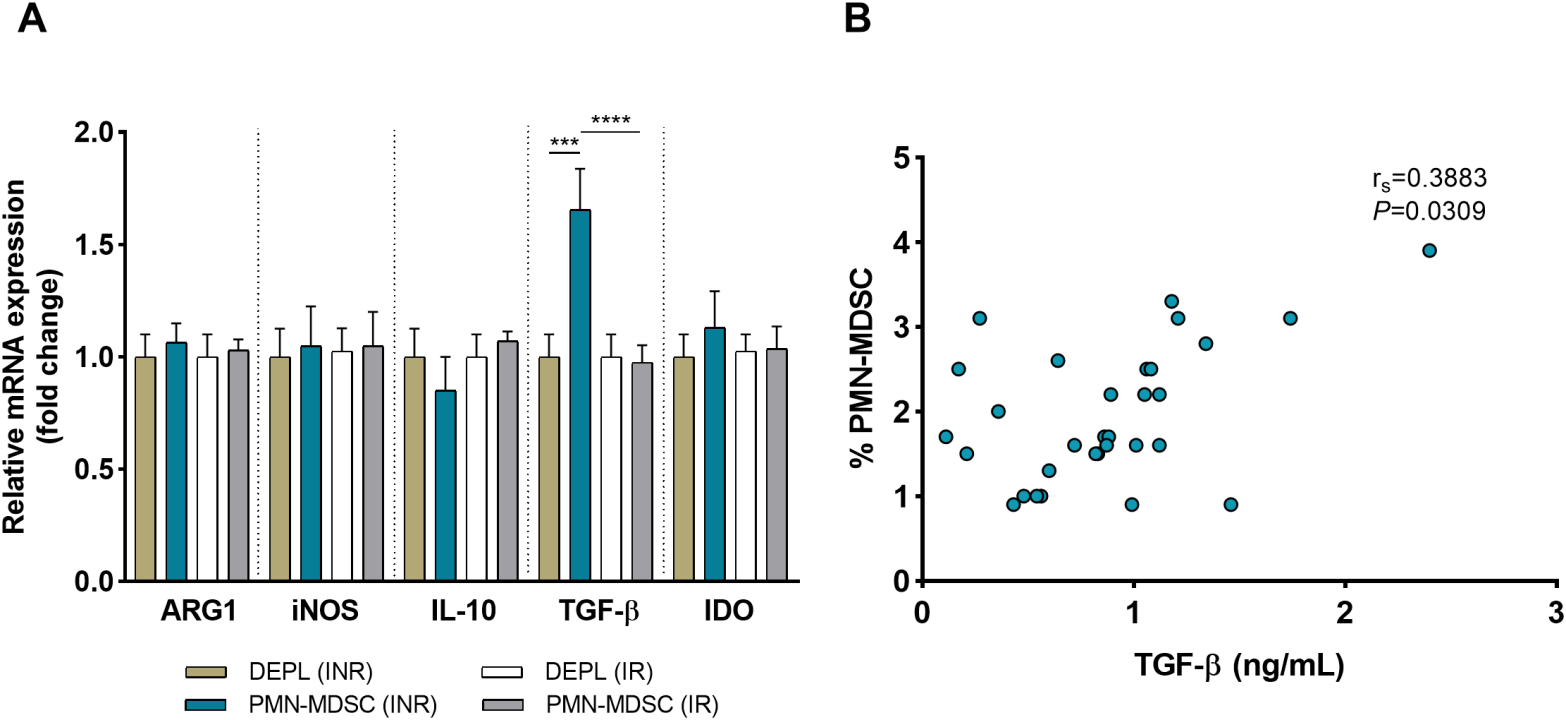
HIV INRs display elevated expression of TGF-β on PMN-MDSCs. **(A)** Comparative analysis on the mRNA expression of ARG1, iNOS, IL-10, TGF-β, and IDO in pure PMN-MDSCs versus DEPL. Data presented are from 10 INRs and 10 IRs. **(B)** Correlation between the frequency of PMN-MDSCs and plasma TGF-β levels in 31 INRs. Non-parametric Spearman correlation test was performed. ***P < 0.001, ****P < 0.0001 (one-way ANOVA). HC, Healthy controls; INR, Immunological non-responders; IR, Immunological responders; ARG1, arginase 1; iNOS, inducible nitric oxide synthase; IL-10, interleukin 10; TGF-β, transforming growth factor beta; IDO, indoleamine 2,3-dioxygenase.

In addition to surface PD-L1, freshly sorted PMN-MDSCs and DEPL from 10 INRs and 10 IRs were examined to assess mRNA expressions of immune regulatory molecules relevant to MDSC activities (ARG1, iNOS, IL-10, TGF-β, and IDO). In INRs and IRs, mRNA from ARG1, iNOS, IL-10, and IDO were expressed at relatively analogous levels in PMN-MDSCs and DEPL (**Figure 4A**) Within the INRs group, the results showed that PMN-MDSCs had much higher levels of TGF-β than DEPL (*P* < 0.001, **Figure 4A**). Compared to IRs, TGF-β mRNA levels were significantly increased in PMN-MDSCs from INRs (*P* < 0.0001). Of note, none of the preceding molecules was upregulated in PMN-MDSCs from IRs. We subsequently examined the levels of TGF-β in plasma from INRs (*n* = 31) and discovered a clear association between PMN-MDSCs and TGF-β (r_s_ = 0.3883, *P* = 0.0309; **Figure 4B**), demonstrating that TGF-β production and release are affected by PMN-MDSCs.

### 3.6 Blocking PD-L1 and TGF-β enhance CD4+ T-cell responses

**Figure 5:**
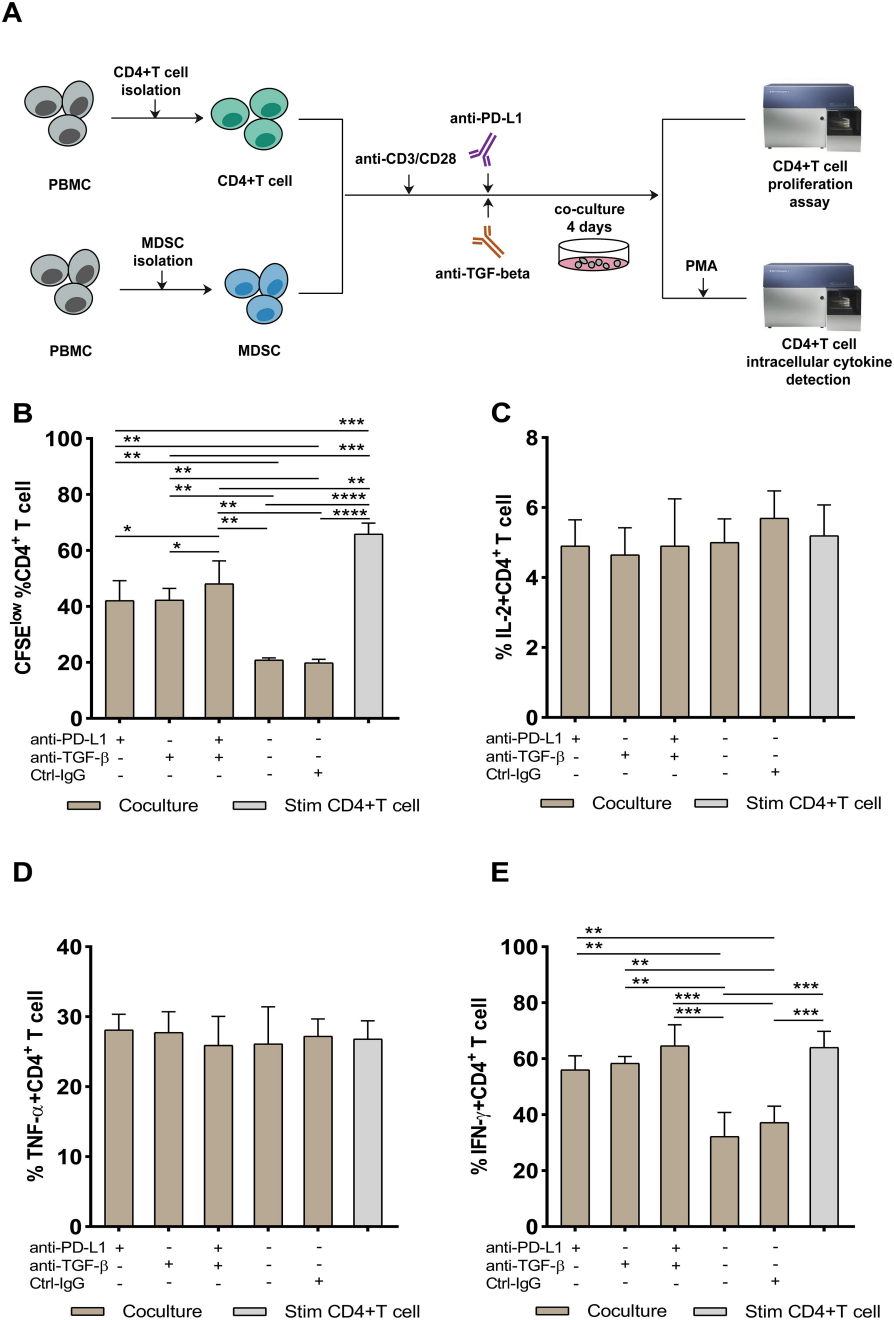
PD-L1 and TGF-β mediate the suppressive effect of PMN-MDSCs on CD4+ T-cells response. Schematic representation of the *in vitro* blocking experiment **(A)**. Effect of blocking either PD-L1, TGF‐β, or both on CD4+ T-cell proliferation **(B)**, IL-2 **(C)**, TNF-α **(D)**, and IFN-γ **(E)** secretion. PMN-MDSCs and CD4+ T cells are from 10 INRs. Stimulated CD4+ T-cells were used as positive control. HC, Healthy controls; INR, Immunological non-responders; IR, Immunological responders. *P ≤ 0.05, **P ≤ 0.01, ***P < 0.001 (one-way ANOVA).

Further investigations were undertaken to analyze the effects of the blockade of PD-L1 and TGF-β signaling on MDSCs-induced suppression of CD4+ T-cell responses. In the presence of anti-CD3/anti-CD28 beads, purified CD4+ T-cells from INRs were cocultured with autologous PMN-MDSCs at different ratios (2:1, 4:1, 8:1). Autologous CD4+ T-cell proliferation was similarly inhibited by PMN-MDSCs in a dose-independent manner (**Supplementary Figure 2**). Inhibitors/antibodies targeting PD-L1, TGF-β, or control antibodies were added. A schematic representation of the experiment procedure is shown in **Figure 5A**. As shown in **Figures 5B** and **E**, we discovered that PD-L1 neutralizing antibodies, TGF- antibodies, and dual PD-L1 and TGF-β blocking greatly increased CD4+ T-cell proliferation and IFN-γ secreting capacity. However, there was no increase in the frequency of CD4+ T-cells producing IL-2 and TNF-α (**Figures 5C, D**). Notably, a simultaneous blockade of both, PD-L1 and TGF-β had a greater impact on reviving CD4+ T-cells proliferation than either anti-PD-L1 or anti-TGF-β therapy alone (**Figure 5B**). Furthermore, inhibiting both PD-L1 and TGF-β were observed to relatively enhance IFN-γ production (**Figure 5E**). Our results suggest that to reverse the inhibitory activities of MDSCs during HIV-1 infection, an optimal strategy should simultaneously target both TGF-β and PD-L1 pathways.

## 4 Discussion

Although the immunosuppressive role of MDSCs is well established, their identification and particular functions in HIV individuals’ immunological recovery remain unknown. Herein, we demonstrated that in INRs, the proportions of MDSCs were higher than in IRs. MDSCs levels were negatively correlated with CD4 counts, suggesting that MDSCs could contribute to immunological recovery. MDSCs may decrease CD4 responses through the PD-L1 and TGF-β pathways.

Distinct MDSC-subsets and various immunosuppressive mechanisms have been reported in chronic viral infection as well as in various cancers (Pal et al., 2019; Yaseen et al., 2021; Ma et al., 2022; Pal et al., 2022). Inconsistent results regarding which subset of MDSCs is expanded during HIV-1 infection were reported in many studies. In line with previous research (Bowers et al., 2014; Tumino et al., 2015), our findings showed that compared to M-MDSC populations, circulating PMN-MDSCs are much more predominant during chronic HIV-1 infection. However, it was also shown by Wang et al (Wang et al., 2016), Garg et al (Garg and Spector, 2014), and Qin et al (Qin et al., 2013). that M-MDSC levels are elevated during the chronic phase of HIV-1 infection. These differences in MDSC (subset) frequencies might be partially explained by differences in the phenotypic markers used to determine MDSCs or by utilizing various sample preparation procedures (Yaseen et al., 2021).

The suppressive effects of MDSCs are mediated via a multitude of concomitant mechanisms, which might vary based on the disease. Previous findings have shown that elevated ARG1 or iNOS production may explain their suppressive mechanism during HIV-1 infection (Garg and Spector, 2014; Yaseen et al., 2021). We mainly focused on five immunosuppressive mediators produced by MDSCs that are known to dampen T-cell responses predominantly in cancer settings and evaluated which of these mediators were utilized by MDSCs in INRs. Our study showed that TGF-β upregulation indicates its implication in the inadequate immune recovery observed in INRs. TGF-β was reported to be a significant pathway used by MDSCs in several human illnesses, e.g., type 1 diabetes, lung cancer, and SARS-CoV-2 (Grohova et al., 2020; Sacchi et al., 2020; Mojsilovic et al., 2022). Thus, researchers postulated that TGF-β may be the most important regulator of immunological responses (Kulkarni et al., 1993) as blockade of TGF-β signaling enhanced simian immunodeficiency virus (SIV)-specific T-cell responses (Samer et al., 2022).

In this study, we found that utilization of inhibitors/neutralizing antibodies targeting TGF-β or PD-L1 abolished MDSCs inhibiting effects and restored CD4+ T-cell functions. By binding PD-L1 to PD-1 on T-cells, MDSCs inhibit T-cells activation and cause apoptosis in the field of tumor research (Noman et al., 2014; Lu et al., 2016a). In HIV individuals, PD-L1 expression on MDSCs has been reported to be correlated to PD-1 expression on CD8+ T-cells (Zhang et al., 2017). IFN-γ production by MDSC-inhibited CD8+ T-cells was dramatically recovered by PD-L1 blockade. Likewise, since blocking the PD-1/PD-L1 axis significantly restored the PMN-MDSC-inhibited CD4+ T-cell function, our findings suggest that the PD-1/PD-L1 axis contributes to the suppressive activities of MDSCs in the immune recovery process. In addition, we observed that strong CD4 response was induced by the administration of an anti-PD-L1 antibody and a TGF-β inhibitor simultaneously, indicating that both PD-L1 and TGF-β are critical components of the T-cell compartment’s signaling pathway. Similarly, in cancer research, the bifunctional inhibition of PD-1/PD-L1 and TGF-β pathways is a novel approach to enhance antitumor effectiveness (Holmgaard et al., 2018; Wu et al., 2022).

T-cell proliferation and cytokine release tests are recognized standard experiments used to evaluate MDSCs suppressive activity (Bruger et al., 2019). The most often examined effector cytokine is IFN-γ. In our investigative study, the production of Th1 cytokines was assessed using flow cytometry. Interestingly, the addition of MDSCs inhibited autologous CD4+ T-cell production of IFN-γ but not the other measured cytokines. Our results are consistent with those of a previous work by Samer et al., which showed that blocking TGF-β1 enhanced SIV-specific CD4+ T-cell IFN-γ expression (Samer et al., 2022). Our study provides evidence that PMN-MDSCs contribute to CD4+ T-cell dysfunction, particularly intercellular IFN-γ secretion in INRs.

This study has several limitations. Firstly, for functional experiments, MDSC suppression of T-cell responses were measured using PMA/ionomycin T-cell stimulation, cytokine production should be assessed using HIV-specific antigen stimulation to further investigate the probable MDSC suppressive mechanism. Secondarily, the cross-sectional study design does not allow us to assess immunoregulatory status over time. Finally, INR and IR groups were not matched on nadir CD4+ T-cell counts as seen in previous studies (Lu et al., 2016b; Luo et al., 2017). Yet, it is known that nadir CD4 are related to inadequate CD4+ T-cell recovery (Kroeze et al., 2018). Consequently, the difference observed in our study may be influenced by this selection bias. Unfortunately, we failed to clear this selection bias as it is challenging to recruit an adequate number of nadir CD4+ T-cell-matching HIV subjects with different immunological response to ART.

In conclusion, the current study demonstrated that PMN-MDSCs expansion was associated with poor CD4 recovery. Furthermore, this study showed that both PD-L1 and TGF-β pathways were involved in MDSCs function. Understanding the properties of MDSCs during HIV-1 infection is necessary to develop new therapeutic strategies.

## Data Availability

The raw data supporting the conclusions of this article will be made available by the authors, without undue reservation.

## References

[B1] Ademe M. (2020). Paradoxes in the phenotype, frequency and roles of myeloid-derived suppressor cells during HIV infection. HIV AIDS (Auckl) 12, 151–156. doi: 10.2147/HIV.S248642 32341663 PMC7166052

[B2] Bowers N. L. Helton E. S. Huijbregts R. P. Goepfert P. A. Heath S. L. Hel Z. (2014). Immune suppression by neutrophils in HIV-1 infection: role of PD-L1/PD-1 pathway. PloS Pathog. 10, e1003993. doi: 10.1371/journal.ppat.1003993 24626392 PMC3953441

[B3] Bronte V. Brandau S. Chen S. H. Colombo M. P. Frey A. B. Greten T. F.. (2016). Recommendations for myeloid-derived suppressor cell nomenclature and characterization standards. Nat. Commun. 7, 12150. doi: 10.1038/ncomms12150 27381735 PMC4935811

[B4] Bruger A. M. Dorhoi A. Esendagli G. Barczyk-Kahlert K. van der Bruggen P. Lipoldova M.. (2019). How to measure the immunosuppressive activity of MDSC: assays, problems and potential solutions. Cancer Immunol. Immunother. 68, 631–644. doi: 10.1007/s00262-018-2170-8 29785656 PMC11028070

[B5] Dorhoi A. Glaria E. Garcia-Tellez T. Nieuwenhuizen N. E. Zelinskyy G. Favier B.. (2019). MDSCs in infectious diseases: regulation, roles, and readjustment. Cancer Immunol. Immunother. 68, 673–685. doi: 10.1007/s00262-018-2277-y 30569204 PMC11028159

[B6] Dross S. E. Munson P. V. Kim S. E. Bratt D. L. Tunggal H. C. Gervassi A. L.. (2017). Kinetics of myeloid-derived suppressor cell frequency and function during simian immunodeficiency virus infection, combination antiretroviral therapy, and treatment interruption. J. Immunol. 198, 757–766. doi: 10.4049/jimmunol.1600759 27974456 PMC5225043

[B7] Gama L. Shirk E. N. Russell J. N. Carvalho K. I. Li M. Queen S. E.. (2012). Expansion of a subset of CD14highCD16negCCR2low/neg monocytes functionally similar to myeloid-derived suppressor cells during SIV and HIV infection. J. Leukoc. Biol. 91, 803–816. doi: 10.1189/jlb.1111579 22368280 PMC3336772

[B8] Garg A. Spector S. A. (2014). HIV type 1 gp120-induced expansion of myeloid derived suppressor cells is dependent on interleukin 6 and suppresses immunity. J. Infect. Dis. 209, 441–451. doi: 10.1093/infdis/jit469 23999600 PMC3883171

[B9] Grohova A. Danova K. Adkins I. Sumnik Z. Petruzelkova L. Obermannova B.. (2020). Myeloid - derived suppressor cells in Type 1 diabetes are an expanded population exhibiting diverse T-cell suppressor mechanisms. PloS One 15, e0242092. doi: 10.1371/journal.pone.0242092 33206686 PMC7673497

[B10] Grutzner E. M. Hoffmann T. Wolf E. Gersbacher E. Neizert A. Stirner R.. (2018). Treatment intensification in HIV-infected patients is associated with reduced frequencies of regulatory T cells. Front. Immunol. 9. doi: 10.3389/fimmu.2018.00811 PMC5936794 29760693

[B11] Hegde S. Leader A. M. Merad M. (2021). MDSC: Markers, development, states, and unaddressed complexity. Immunity 54, 875–884. doi: 10.1016/j.immuni.2021.04.004 33979585 PMC8709560

[B12] Holmgaard R. B. Schaer D. A. Li Y. Castaneda S. P. Murphy M. Y. Xu X.. (2018). Targeting the TGFbeta pathway with galunisertib, a TGFbetaRI small molecule inhibitor, promotes anti-tumor immunity leading to durable, complete responses, as monotherapy and in combination with checkpoint blockade. J. Immunother. Cancer 6, 47. doi: 10.1186/s40425-018-0356-4 29866156 PMC5987416

[B13] Kroeze S. Ondoa P. Kityo C. M. Siwale M. Akanmu S. Wellington M.. (2018). Suboptimal immune recovery during antiretroviral therapy with sustained HIV suppression in sub-Saharan Africa. AIDS 32, 1043–1051. doi: 10.1097/QAD.0000000000001801 29547445

[B14] Kulkarni A. B. Huh C. G. Becker D. Geiser A. Lyght M. Flanders K. C.. (1993). Transforming growth factor beta 1 null mutation in mice causes excessive inflammatory response and early death. Proc. Natl. Acad. Sci. U.S.A. 90, 770–774. doi: 10.1073/pnas.90.2.770 8421714 PMC45747

[B15] Lu C. Redd P. S. Lee J. R. Savage N. Liu K. (2016a). The expression profiles and regulation of PD-L1 in tumor-induced myeloid-derived suppressor cells. Oncoimmunology 5, e1247135. doi: 10.1080/2162402X.2016.1247135 28123883 PMC5214087

[B16] Lu X. Su B. Xia H. Zhang X. Liu Z. Ji Y.. (2016b). Low double-negative CD3(+)CD4(-)CD8(-) T cells are associated with incomplete restoration of CD4(+) T cells and higher immune activation in HIV-1 immunological non-responders. Front. Immunol. 7. doi: 10.3389/fimmu.2016.00579 PMC5145861 28018346

[B17] Luo Z. Li Z. Martin L. Hu Z. Wu H. Wan Z.. (2017). Increased natural killer cell activation in HIV-infected immunologic non-responders correlates with CD4+ T cell recovery after antiretroviral therapy and viral suppression. PloS One 12, e0167640. doi: 10.1371/journal.pone.0167640 28076376 PMC5226712

[B18] Ma T. Renz B. W. Ilmer M. Koch D. Yang Y. Werner J.. (2022). Myeloid-derived suppressor cells in solid tumors. Cells 11, 310. doi: 10.3390/cells11020310 PMC8774531 35053426

[B19] Mojsilovic S. Mojsilovic S. S. Bjelica S. Santibanez J. F. (2022). Transforming growth factor-beta1 and myeloid-derived suppressor cells: A cancerous partnership. Dev. Dyn 251, 105–124. doi: 10.1002/dvdy.339 33797140

[B20] Noman M. Z. Desantis G. Janji B. Hasmim M. Karray S. Dessen P.. (2014). PD-L1 is a novel direct target of HIF-1alpha, and its blockade under hypoxia enhanced MDSC-mediated T cell activation. J. Exp. Med. 211, 781–790. doi: 10.1084/jem.20131916 24778419 PMC4010891

[B21] Nourbakhsh E. Mohammadi A. Salemizadeh Parizi M. Mansouri A. Ebrahimzadeh F. (2021). Role of Myeloid-derived suppressor cell (MDSC) in autoimmunity and its potential as a therapeutic target. Inflammopharmacology 29, 1307–1315. doi: 10.1007/s10787-021-00846-3 34283371

[B22] Ostrand-Rosenberg S. Lamb T. J. Pawelec G. (2023). Here, there, and everywhere: myeloid-derived suppressor cells in immunology. J. Immunol. 210, 1183–1197. doi: 10.4049/jimmunol.2200914 37068300 PMC10111205

[B23] Pal S. Dey D. Chakraborty B. C. Nandi M. Khatun M. Banerjee S.. (2022). Diverse facets of MDSC in different phases of chronic HBV infection: Impact on HBV-specific T-cell response and homing. Hepatology 76, 759–774. doi: 10.1002/hep.32331 35000202

[B24] Pal S. Nandi M. Dey D. Chakraborty B. C. Shil A. Ghosh S.. (2019). Myeloid-derived suppressor cells induce regulatory T cells in chronically HBV infected patients with high levels of hepatitis B surface antigen and persist after antiviral therapy. Aliment Pharmacol. Ther. 49, 1346–1359. doi: 10.1111/apt.15226 30982998

[B25] Pawelec G. Verschoor C. P. Ostrand-Rosenberg S. (2019). Myeloid-derived suppressor cells: not only in tumor immunity. Front. Immunol. 10. doi: 10.3389/fimmu.2019.01099 PMC6529572 31156644

[B26] Qin A. Cai W. Pan T. Wu K. Yang Q. Wang N.. (2013). Expansion of monocytic myeloid-derived suppressor cells dampens T cell function in HIV-1-seropositive individuals. J. Virol. 87, 1477–1490. doi: 10.1128/JVI.01759-12 23152536 PMC3554138

[B27] Sacchi A. Grassi G. Bordoni V. Lorenzini P. Cimini E. Casetti R.. (2020). Early expansion of myeloid-derived suppressor cells inhibits SARS-CoV-2 specific T-cell response and may predict fatal COVID-19 outcome. Cell Death Dis. 11, 921. doi: 10.1038/s41419-020-03125-1 33110074 PMC7590570

[B28] Samer S. Thomas Y. Arainga M. Carter C. Shirreff L. M. Arif M. S.. (2022). Blockade of TGF-beta signaling reactivates HIV-1/SIV reservoirs and immune responses in *vivo* . JCI Insight 7, e162290. doi: 10.1172/jci.insight.162290 36125890 PMC9675457

[B29] Tumino N. Turchi F. Meschi S. Lalle E. Bordoni V. Casetti R.. (2015). In HIV-positive patients, myeloid-derived suppressor cells induce T-cell anergy by suppressing CD3zeta expression through ELF-1 inhibition. AIDS 29, 2397–2407. doi: 10.1097/QAD.0000000000000871 26355672

[B30] Vanhaver C. van der Bruggen P. Bruger A. M. (2021). MDSC in mice and men: mechanisms of immunosuppression in cancer. J. Clin. Med. 10, 2872. doi: 10.3390/jcm10132872 PMC8268873 34203451

[B31] Veglia F. Perego M. Gabrilovich D. (2018). Myeloid-derived suppressor cells coming of age. Nat. Immunol. 19, 108–119. doi: 10.1038/s41590-017-0022-x 29348500 PMC5854158

[B32] Vollbrecht T. Stirner R. Tufman A. Roider J. Huber R. M. Bogner J. R.. (2012). Chronic progressive HIV-1 infection is associated with elevated levels of myeloid-derived suppressor cells. AIDS 26, F31–F37. doi: 10.1097/QAD.0b013e328354b43f 22526518

[B33] Wang L. Zhao J. Ren J. P. Wu X. Y. Morrison Z. D. Elgazzar M. A.. (2016). Expansion of myeloid-derived suppressor cells promotes differentiation of regulatory T cells in HIV-1+ individuals. AIDS 30, 1521–1531. doi: 10.1097/QAD.0000000000001083 26959508 PMC4889474

[B34] Wu Z. H. Li N. Gao Z. Z. Chen G. Nie L. Zhou Y. Q.. (2022). Development of the novel bifunctional fusion protein BR102 that simultaneously targets PD-L1 and TGF-beta for anticancer immunotherapy. Cancers (Basel) 14, 4964. doi: 10.3390/cancers14194964 PMC9562016 36230887

[B35] Xia H. Jiang W. Zhang X. Qin L. Su B. Li Z.. (2018). Elevated level of CD4(+) T cell immune activation in acutely HIV-1-infected stage associates with increased IL-2 production and cycling expression, and subsequent CD4(+) T cell preservation. Front. Immunol. 9. doi: 10.3389/fimmu.2018.00616 PMC5880913 29636753

[B36] Yang X. Su B. Zhang X. Liu Y. Wu H. Zhang T. (2020). Incomplete immune reconstitution in HIV/AIDS patients on antiretroviral therapy: Challenges of immunological non-responders. J. Leukoc. Biol. 107, 597–612. doi: 10.1002/JLB.4MR1019-189R 31965635 PMC7187275

[B37] Yaseen M. M. Abuharfeil N. M. Darmani H. (2021). Myeloid-derived suppressor cells and the pathogenesis of human immunodeficiency virus infection. Open Biol. 11, 210216. doi: 10.1098/rsob.210216 34753323 PMC8580465

[B38] Yi M. Zheng X. Niu M. Zhu S. Ge H. Wu K. (2022). Combination strategies with PD-1/PD-L1 blockade: current advances and future directions. Mol. Cancer 21, 28. doi: 10.1186/s12943-021-01489-2 35062949 PMC8780712

[B39] Zhang Z. N. Yi N. Zhang T. W. Zhang L. L. Wu X. Liu M.. (2017). Myeloid-derived suppressor cells associated with disease progression in primary HIV infection: PD-L1 blockade attenuates inhibition. J. Acquir. Immune Defic. Syndr. 76, 200–208. doi: 10.1097/QAI.0000000000001471 28570288

