# Supplementary material for: Polymorphonuclear myeloid-derived suppressor cells regulates immune recovery during HIV infection through PD-L1 and TGF-β pathways
Source: Front Cell Infect Microbiol. 2024 Dec 17;14:1516421. doi: 10.3389/fcimb.2024.1516421 (PMC11685070; doi:10.3389/fcimb.2024.1516421)
Supplement: Supplementary file 1 [file Presentation1.pptx]

## Slide 1
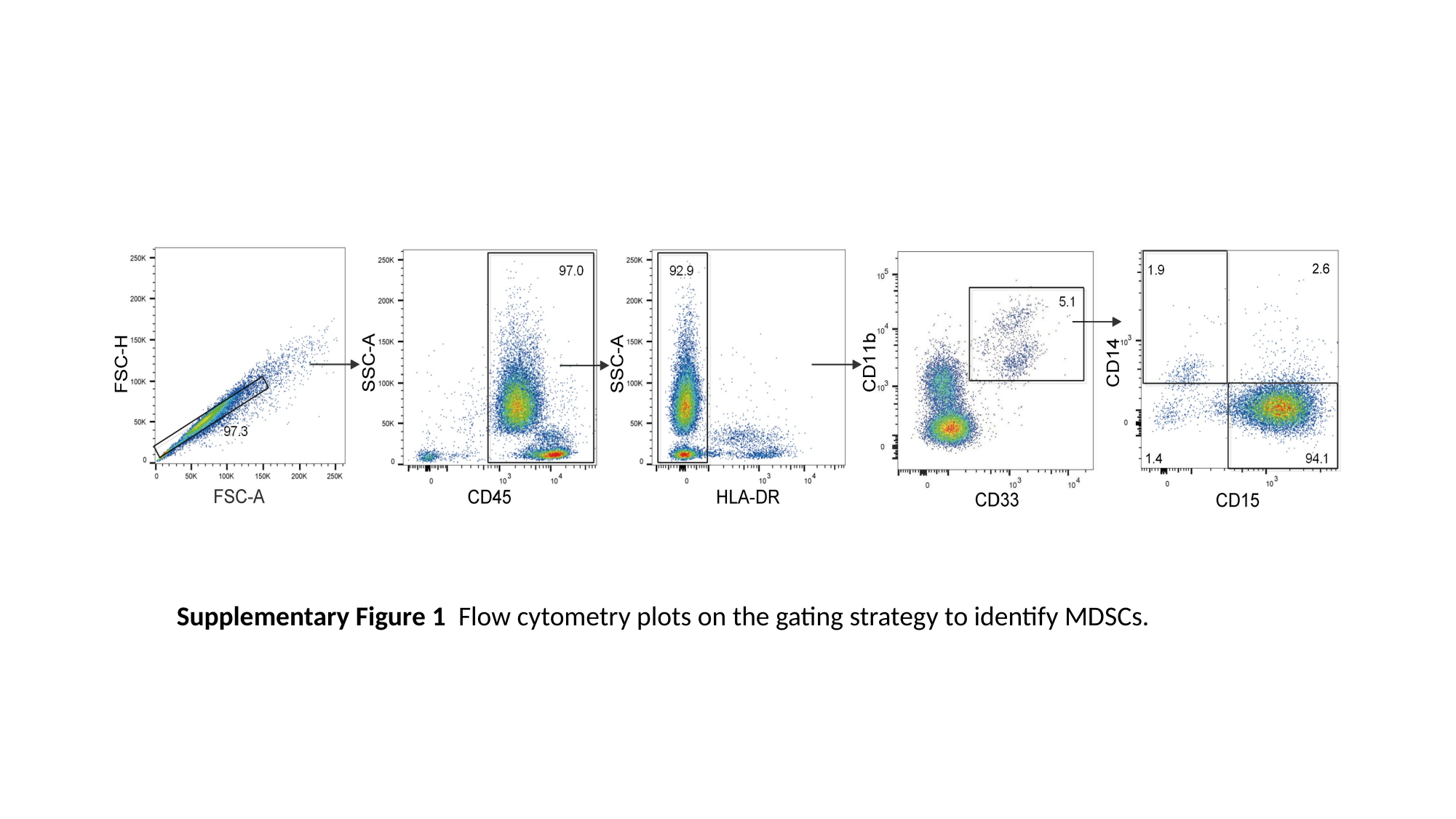

Supplementary Figure 1 Flow cytometry plots on the gating strategy to identify MDSCs.

## Slide 2
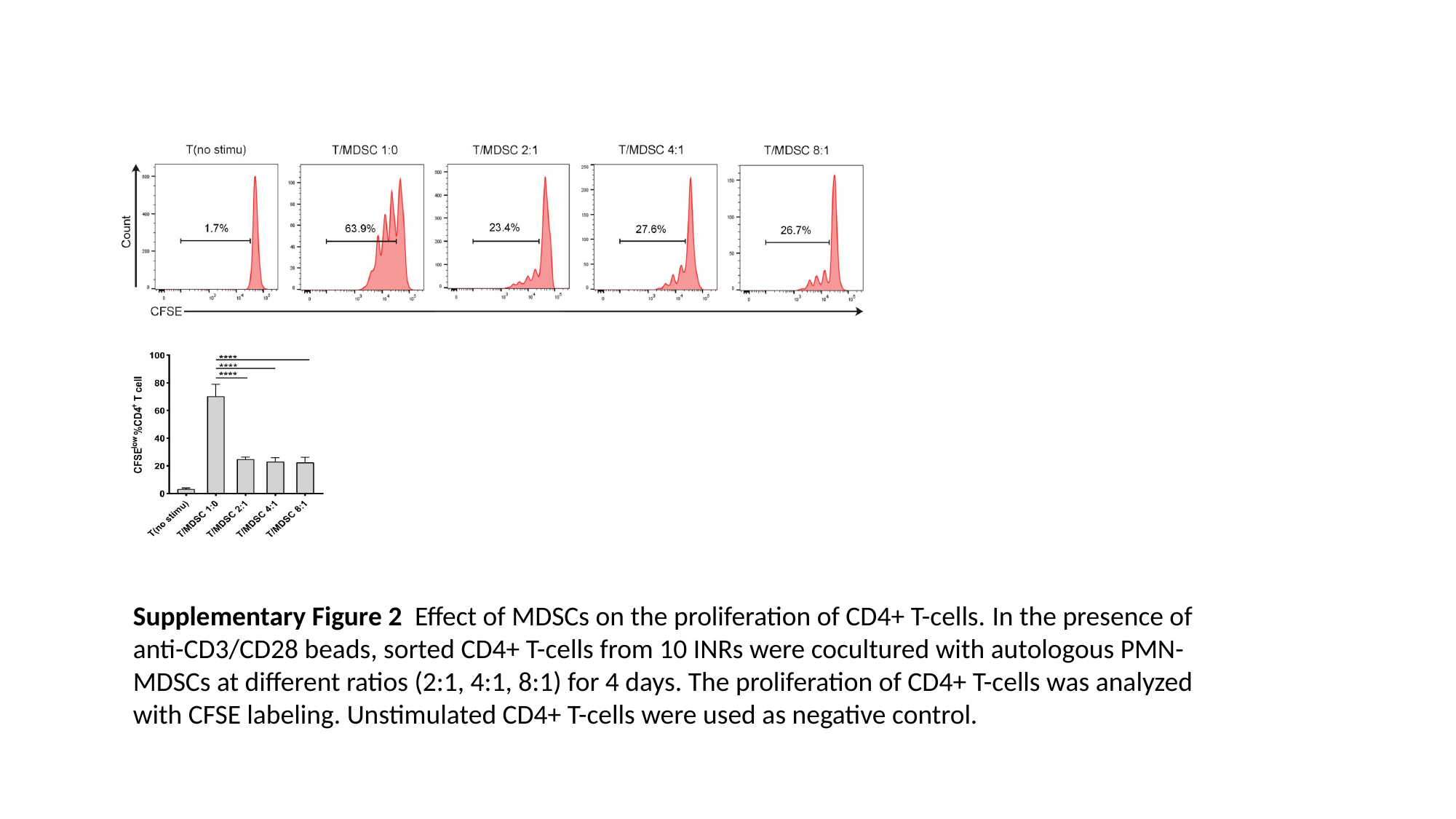

Supplementary Figure 2 Effect of MDSCs on the proliferation of CD4+ T-cells. In the presence of anti-CD3/CD28 beads, sorted CD4+ T-cells from 10 INRs were cocultured with autologous PMN-MDSCs at different ratios (2:1, 4:1, 8:1) for 4 days. The proliferation of CD4+ T-cells was analyzed with CFSE labeling. Unstimulated CD4+ T-cells were used as negative control.
